# Quantitative muscle MRI to follow up late onset Pompe patients: a prospective study

**DOI:** 10.1038/s41598-018-29170-7

**Published:** 2018-07-18

**Authors:** Sebastian Figueroa-Bonaparte, Jaume Llauger, Sonia Segovia, Izaskun Belmonte, Irene Pedrosa, Elena Montiel, Paula Montesinos, Javier Sánchez-González, Alicia Alonso-Jiménez, Eduard Gallardo, Isabel Illa, Spanish Pompe group, Miguel Angel Barba-Romero, Joseba Barcena, Pilar Carbonell, María Rosario Carzorla, Carlota Creus, Jaume Coll-Cantí, Manuel Díaz, Cristina Domínguez, Roberto Fernández-Torrón, María José García-Antelo, Josep Maria Grau, Adolfo López de Munáin, Francisco Antonio Martínez-García, Yolanda Morgado, Antonio Moreno, Germán Morís, Miguel Angel Muñoz-Blanco, Andres Nascimento, José Luis Parajua, Arturo Robledo-Strauss, Íñigo Rojas-Marcos, Jose António Salazar, Mercedes Usón, Jordi Díaz-Manera

**Affiliations:** 1 Neuromuscular Disorders Unit, Neurology Department, Hospital de la Santa Creu i Sant Pau, Universitat Autònoma de Barcelona, Barcelona, Spain; 2 Centro de Investigación en Red en Enfermedades Raras (CIBERER), Madrid, Spain; 3 Radiology department, Hospital de la Santa Creu i Sant Pau, Universitat Autònoma de Barcelona, Barcelona, Spain; 4 Rehabilitation and physiotherapy department, Hospital de la Santa Creu i Sant Pau, Universitat Autònoma de Barcelona, Barcelona, Spain; 5 Philips Healthcare Iberia, Madrid, Spain; 6 Internal Medicine Department, Hospital General de Albacete, Albacete, Spain; 7 Neurology department, Hospital Universitario Cruces, Baracaldo, Spain; 8 Neurology department, Hospital Virgen del Rocío, Sevilla, Spain; 9 Pediatry Department, Hospital Puerta de Hierro, Majadahonda, Spain; 10 Neurology Department, Hospital Virgen de las Nieves, Granada, Spain; 11 Neurology Department, Hospital Germans Tries i Pujol, Badalona, Spain; 12 Neurology Department, Hospital de Cabueñes, Gijón, Spain; 13 Neurology Department, Hospital 12 de Octubre, Madrid, Spain; 14 Neurology Department, Hospital Universitario Donostia, Donostia, Spain; 15 Neurology Department, Hospital Universitario A Coruña, A Coruña, Spain; 16 Internal Medicine Department, Hospital Clínic, Barcelona, Spain; 17 Neurology department, Hospital Clínico Universitario Virgen de la Arrixaca, Murcia, Spain; 18 Neurology Department, Hospital Universitario Virgen de Valme, Sevilla, Spain; 19 Neurology Department, Hospital Universitario Morales Meseguer, Murcia, Spain; 20 Neurology Department, Hospital Universitario de Asturias, Oviedo, Spain; 21 Neurology Department, Hospital Gregorio Marañón, Madrid, Spain; 22 Pediatry Department, Hospital Sant Joan de Déu, Barcelona, Spain; 23 Internal Medicine Department, Hospital de Can Mises, Ibiza, Spain; 24 Neurology Department, Hospital Juan Ramón Jiménez, Huelva, Spain; 25 Neurology Department, Hospital Virgen de Macarena, Sevilla, Spain; 26 Neurology Department, Hospital Regional Universitario de Málaga, Málaga, Spain; 27 Neurology Department, Hospital de Son Llátzer, Palma de Mallorca, Balearic Islands, Spain

## Abstract

Late onset Pompe disease (LOPD) is a slow, progressive disorder characterized by skeletal and respiratory muscle weakness. Enzyme replacement therapy (ERT) slows down the progression of muscle symptoms. Reliable biomarkers are needed to follow up ERT-treated and asymptomatic LOPD patients in clinical practice. In this study, 32 LOPD patients (22 symptomatic and 10 asymptomatic) underwent muscle MRI using 3-point Dixon and were evaluated at the time of the MRI with several motor function tests and patient-reported outcome measures, and again after one year. Muscle MRI showed a significant increase of 1.7% in the fat content of the thigh muscles in symptomatic LOPD patients. In contrast, there were no noteworthy differences between muscle function tests in the same period of time. We did not observe any significant changes either in muscle MRI or in muscle function tests in asymptomatic patients over the year. We conclude that 3-point Dixon muscle MRI is a useful tool for detecting changes in muscle structure in symptomatic LOPD patients and could become part of the current follow-up protocol in daily clinics.

## Introduction

Pompe disease is a genetic disorder characterized by glycogen accumulation in all tissues of the body^1^. Pompe patients are classified as infantile or late onset (LOPD) depending on their age when symptoms first appear. LOPD patients have variable clinical presentations, ranging from asymptomatic hyperCKemia to limb girdle and respiratory muscle weakness^2^. Enzymatic replacement therapy (ERT) is indicated for LOPD patients with skeletal muscle weakness and/or respiratory involvement^3^. Several studies have reported motor and respiratory stabilization during the first few years of ERT, and even a decrease in the mortality rate^4–9^. However, it has been suggested that ERT does not stop muscle degeneration at the histological level^10^. Indeed, recently published data have shown impairment in muscle and respiratory function after several years of treatment^11^.

In following up asymptomatic LOPD patients, the main aim is to detect changes in muscle function that could support ERT treatment. However, normal muscle function tests do not reveal the integrity of the muscle structure of these patients; muscle fiber loss and fatty replacement could have started without yet influencing the results of the tests. Moreover, it is questionable whether muscle function tests are precise enough to detect subtle changes, and most authors agree that we need reliable non-invasive biomarkers of disease progression^12,13^.

Quantitative muscle MRI (qMRI) has emerged as a valuable biomarker to follow up the progression of neuromuscular disorders^14–18^. qMRI is a non-invasive tool that quantifies the amount of fat present in a muscle’s region of interest (ROI). The total muscle area and remaining muscle tissue can also be calculated^16,17^. We have previously demonstrated that qMRI correlates strongly with common outcome measures used in LOPD^19^. Our main aim was to investigate MRI changes occurring in the muscles of LOPD patients and assess whether qMRI was more sensitive to changes after one year than other commonly used motor function tests or patient-reported outcome measures (PROMs).

## Results

### Description of the cohort

We recruited 32 late onset Pompe disease (LOPD) patients for this study. They were classified as symptomatic or asymptomatic depending on the presence of weakness at clinical examination. Twenty-two patients were symptomatic and treated with ERT while ten were non-symptomatic and only had hyperCKemia. This second group was studied in neuromuscular disorder units because high levels of CKs were found in routine blood tests (7 cases) or because they had relatives already diagnosed with Pompe disease (3 cases). Clinical and genetic data of the patients included in the study are described in Tables 1 and 2. We found two pathogenic mutations in all our patients, except for patient 29. That patient, in absence of defined GAA variants, was diagnosed by biochemical results as GAA deficieny in leucocytes with the values of 0.03 nmol/min/mg protein (normal values: 0.15–1 nmol/min/mg protein) and confirmed in fibroblasts with the value of 21.4 nmol/min/mg protein (normal values 400–600 nmol/min/mg protein).

**Table 1:** Clinical characteristics of the cohort of LOPD patients participating in the study.

N	Gender	Age at study (y)	Phenotype	Mut 1	Mut 2	CK (U/l)	ERT	Age at ERT	Wheelchair dependent	Respiratory support
1	Female	50	Proximal weakness LL + axial	IVS1-13T > G	c.1076-1 G > C	251	Yes	47	N	N
2	Female	48	Proximal weakness UL and LL + axial + respiratory insufficiency	IVS1-13T > G	c.2173 C > T	779	Yes	39	Y	NIV
3	Female	26	HyperCKemia	IVS1-13T > G	c.1889-1 G > A	720	No	−	N	N
4	Female	63	Proximal weakness LL + axial	IVS1-13T > G	c.2600_2604delinsA	311	Yes	59	N	N
5	Male	11	HyperCKemia	IVS1-13T > G	c.573 > A	276	No	—	N	N
6	Female	45	Proximal weakness LL	IVS1-13T > G	c.1532 C > A	322	Yes	42	N	N
7	Female	51	Proximal weakness LL	IVS1-13T > G	c.236_246del	240	Yes	47	N	N
8	Female	59	Proximal weakness LL	IVS1-13T > G	c.1637A > G	341	Yes	52	N	N
9	Female	55	Proximal weakness LL	IVS1-13T > G	c.2173 C > T	359	Yes	48	N	N
10	Male	42	Proximal weakness LL + axial + respiratory insufficiency	IVS1-13T > G	c.573 C > A	606	Yes	39	N	NIV
11	Female	31	Proximal weakness UL and LL + respiratory insufficiency	IVS1-13T > G	c.1637A > G	391	Yes	24	Y	IV
12	Male	47	Proximal weakness LL + respiratory insufficiency	c.2173 C > T	c.2173 C > T	508	Yes	45	N	NIV
13	Male	51	Proximal weakness LL + respiratory insufficiency	IVS1-13T > G	c.1657C > T	709	Yes	45	N	NIV
14	Female	51	Proximal weakness UL and LL + respiratory insufficiency	IVS1-13T > G	c.1657C > T	458	Yes	46	N	NIV
15	Male	22	HyperCKemia	IVS1-13T > G	c.1781G > A	1268	No	—	N	N
16	Male	49	HyperCKemia	c.271 G > A	c.2510 G > A	641	No	—	N	N
17	Male	14	HyperCKemia	IVS1-13T > G	c.573 C > A	660	No	—	N	N
18	Female	65	Proximal weakness LL + respiratory insufficiency	c.1781G > A	c. 1194 + 5 G > A	68	Yes	64	N	N
19	Female	35	Proximal weakness LL	IVS1-13T > G	c.1 A > T	355	Yes	29	N	N
20	Female	40	Proximal weakness LL	IVS1-13T > G	c.1889-1 G > A	831	Yes	—	N	NIV
21	Female	52	Proximal weakness LL + respiratory insufficiency	c.1781G > A	c.1194 + 5 G > A	907	Yes	45	N	N
22	Male	64	Proximal weakness UL + LL + axial + respiratory insufficiency	IVS1-13T > G	c.2481 + 102_2646 + 31del	430	Yes	57	N	NIV
23	Male	8	HyperCKemia	IVS1-13T > G	c.1889-1 G > A	1077	No	—	N	N
24	Female	57	Proximal weakness LL + respiratory insufficiency	IVS1-13T > G	c. 1447 G > T	394	Yes	55	N	NIV
25	Male	46	Proximal weakness LL	IVS1-13T > G	c. 1532 C > A	882	Yes	43	N	NIV
26	Male	51	Proximal weakness LL	IVS1-13T > G	c. 1933G > T	952	Yes	51	N	NIV
27	Male	51	HyperCKemia	IVS1-13T > G	c. 1933G > T	432	No	—	N	N
28	Male	43	Proximal weakness LL	IVS1-13T > G	c. 1408_1410delinsTTT	317	Yes	43	N	N
29	Female	54	Proximal UL and axial weakness	Not found	Not found	275	Yes	48	N	N
30	Female	20	HyperCKemia	IVS1-13T > G	c. 1551 + 1 G > A	928	No	—	N	N
31	Male	50	HyperCKemia	IVS1-13T > G	c. 1551 + 1 G > A	250	No	—	N	N
32	Female	36	HyperCKemia	IVS1-13T > G	IVS1-13T > G	230	No	—	N	N

Normal CK levels were lower than 170 U/L. Patients 13 and 14 and patients 26 and 27 were siblings. Respiratory support: NIV means non-invasive ventilation while IV means invasive ventilation. UL: Upper limbs, LL: lower limbs, Y: yes, N: No.

**Table 2:** Demographic and clinical data of symptomatic and asymptomatic Pompe patients included in the study.

	Patients		
	ERT treated	HyperCKemia	p*
Number of patients	22	10	
Gender (W)	15, 68.2%	3, 30%	0.06
Age at baseline	51 (45–55)	24 (13.5–49.5)	0.04
Time from onset of symptoms	15 (11–22)	—	
Time on ERT	4(2–7)	—	
Walking aids	10	—	
Ventilation	11	—	
Time to walk 10 meters (s)	7.1 (5.3–8.8)	3.1 (2.7–3.4)	0.001
6 MWT (m)	414.5 (306.2–493.2)	603 (535.2–677.7)	0.0001
Time to climb 4 steps (s)	3.4 (2.3–6.5)	1.5 (1.2-1.9)	0.005
Time to descend 4 steps (s)	2.8 (2.1–4.8)	1.4 (1.1–1.6)	0.007
Timed up & go test (s)	5.7 (3.1–7.9)	4 (2.9–5.4)	0.03
MRC	96 (84–106)	120 (118–120)	0.0001
Myometry	141 (101–231)	342 (215–491)	0.02
MFM-20 (score)	47.5 (43.5–55)	59.5 (57.5–60)	0.0001
CVF seated (%)	79.9 (60.2–88.7)	92 (85.8–103)	0.03
CVF lying (%)	68.5 (39.5–81.7)	85.5 (77.5–92.2)	0.02
Activlim (score)	21 (18–26)	18 (18–24)	>0.05
SF36 (score)	56.9 (43.7–69.9)	85.5 (69–90)	0.02
INQoL (score)	39 (23.5–53.9)	2.5 (0.9–18.1)	0.05

Median value and 25^th^–75^th^ percentiles are shown for every variable. S: seconds, m: meters. Mann-Whitney U test was used to compare the groups, except for sex that was studied using Chi square.

### Baseline Muscle MRI and muscle function analysis

**Figure 1:**
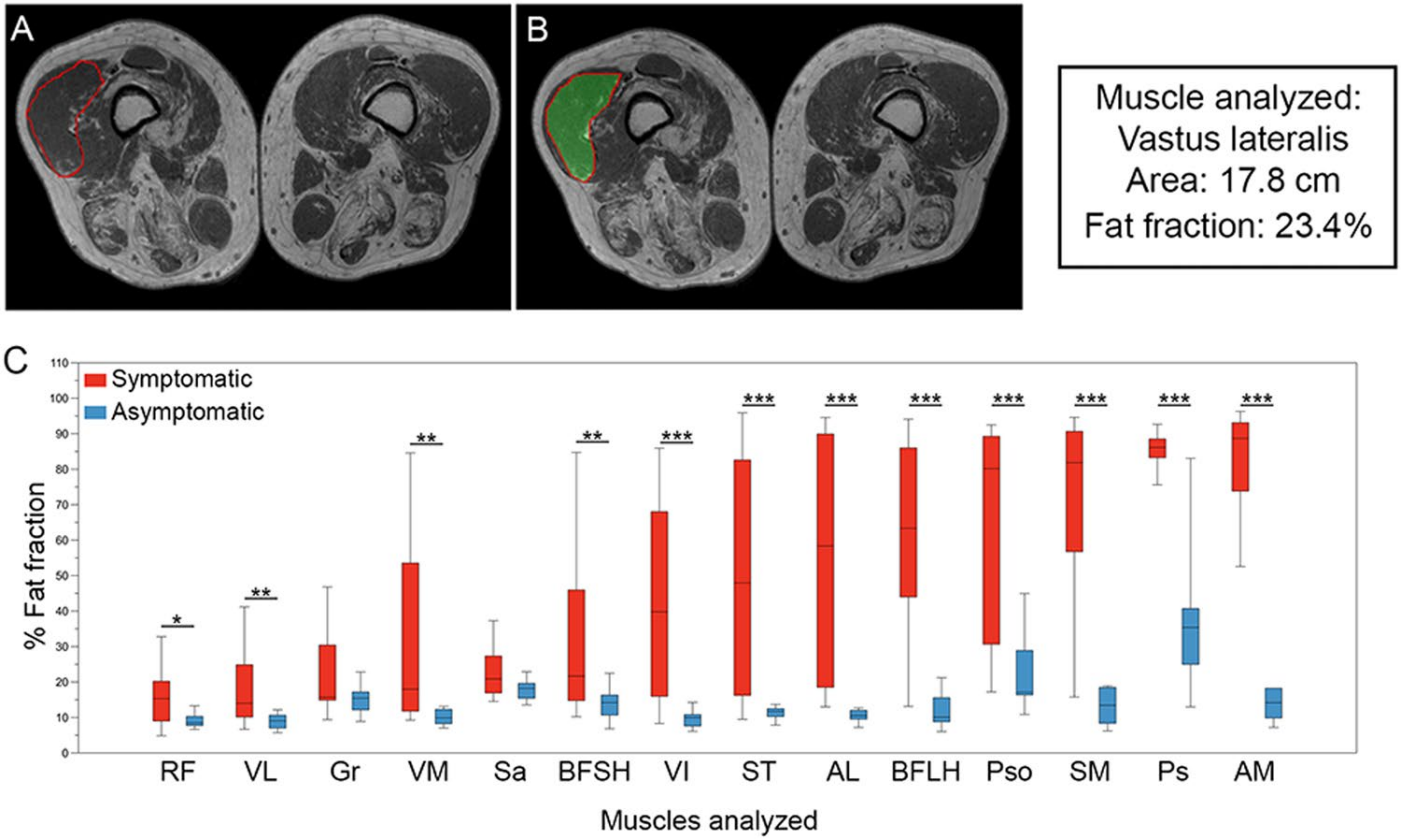
Analysis of fat fraction in thigh and trunk muscles in LOPD patients at baseline. (**A**,**B**) Show an example of how ROIs are drawn to obtain total muscle area and fat fraction in 3-point Dixon images. (**C**) shows the fat fraction calculated for thigh and trunk muscles in symptomatic (red) and asymptomatic (blue) patients. The box plot includes the 25^th^–75^th^ percentile, the mid lines indicate the median, bars are the 5^th^–95^th^ percentiles. *P < 0.05, **P < 0.01 and ***P < 0.001. RF: *Rectus Femoris*, VL: *Vastus Lateralis*, Gr: *Gracilis*, VM: *Vastus Medialis*, Sa: *Sartorius*, BFSH: *Biceps Femoris Short Head*, VI: *Vastus Intermedius*, ST: *Semitendinosus*, AL: *Adductor Longus*, BFLH: *Biceps Femoris Long Head*, Pso: *Psoas*, SM: *Semimembranosus*, Ps: *Paraspinalis*, and AM: *Adductor Major*.

We focused our attention on the trunk and thigh regions because these muscles are the most commonly involved in LOPD^19,20^. Analysis of 3-point Dixon studies showed a higher statistical significance for the fat fraction (FF) in symptomatic compared to asymptomatic LOPD patients in all analyzed muscles, except for *sartorius* and *gracillis* (Fig. 1C).

Thigh FF was significantly higher in symptomatic than asymptomatic patients (Median: 34.03 vs. 11.07, Mann Whitney U test, p = 0.0001). We found strong correlations between muscle function tests and average thigh FF at baseline (Table 3). For example, thigh FF correlated with the total MRC lower limb score (p = 0.0001, ρ = −0.89) or 6MWT results (p = 0.0001, ρ = −0.68). In contrast, there was a poor correlation between thigh FF and respiratory parameters (CVF while seated: p = 0.02, ρ = −0.4; CVF while lying down: p = 0.17, ρ = −0.34), as well as with the results of the patient-reported outcome measures (INQoL: p = 0.05, ρ = 0.37). We did not find a strong correlation between thigh FF and clinical parameters such as age at baseline (p = 0.03, ρ = 0.48), time from onset of symptoms (p = 0.06, ρ = 0.32), or time on ERT (p = 0.013, ρ = 0.52).

**Table 3:** Correlation between Thigh fat fraction and muscle function tests, spirometry results and patient-reported outcomes at baseline visit. Spearman test was used to study whether there was a significant correlation between variables.

Muscle function test	p	Spearman Correlation coefficient
**Muscle function tests**		
MRC score (all muscles)	0.0001	−0.89
MRC score (lower limbs)	0.0001	−0.91
Myometry score (all muscles)	0.0001	−0.64
Myometry score (lower limbs)	0.0001	−0.65
6MWT	0.0001	−0.68
Time to walk 10 meters	0.0001	0.80
Time to climb 4 steps	0.0001	0.84
Time to descend 4 steps	0.0001	0.75
Timed up & go test	*0*.*021*	0.40
MFM-20	0.0001	−0.75
**Spirometry**		
CVF seated	*0*.*022*	−0.40
CVF lying	*0*.*17*	
**Patient-reported outcomes**		
Activlim	*0*.*01*	0.43
SF36 total	*0*.*08*	
SF36 physical	*0*.*004*	−0.53
SF36 mental	*0*.*86*	
INQoL	*0*.*05*	0.37

Adjustment for multiple comparisons using Bonferroni correction was applied; p was considered significant if lower than 0.003.

### One year muscle MRI and muscle function analysis

**Figure 2:**
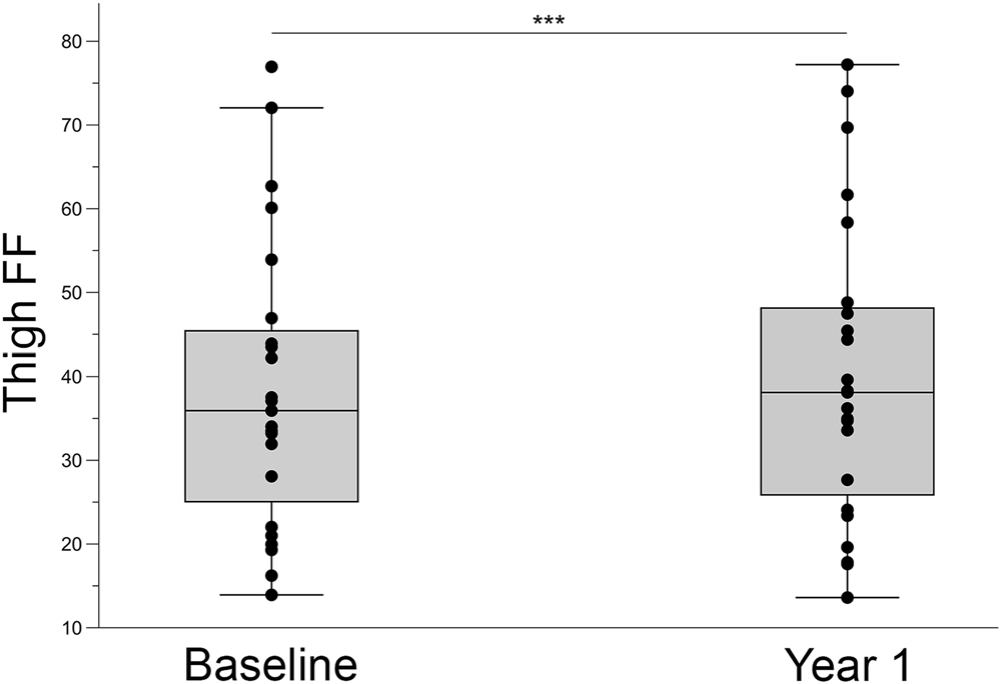
Yearly progression in thigh muscle fat replacement in symptomatic LOPD patients. The box plot includes the 25^th^–75^th^ percentile, the mid lines indicate the median, bars are the 5^th^–95^th^ percentiles. Each dot represents one symptomatic LOPD patient. Mann-Whitney U Test, ***P < 0.001.

We repeated muscle function tests, spirometry, patient-reported outcome measures and muscle MRI for all patients after one year. In symptomatic patients, muscle function tests, spirometry and patient-reported outcomes did not show significant changes from baseline to year 1 (Table 4). In contrast, 3-point Dixon images detected a median increase of 1.7% in thigh FF that was statistically significant (paired Wilcoxon signed rank test, p = 0.001) (Fig. 2). Changes in FF between baseline and year 1 for individual muscles are specified in Table 5.

**Table 4:** Change between baseline and year 1 evaluation in muscle function tests, spirometry, quantitative muscle MRI and patient reported outcome measures in symptomatic and asymptomatic LOPD patients.

	Symptomatic treated patients (baseline vs. 12-month follow-up) (n = 22)	*P* value	Asymptomatic patients (baseline vs. 12-month follow-up) (n = 10)	*P* value
**Muscle function tests**				
MRC total, score	0 (−4; +6)	0.79	0 (0; +1)	0.31
MRC Lower limbs, score	0 (−5; +4)	0.54	0 (0)	0.31
Knee extension (Nm)	10.74 (0; +20.44)	0.19	−0.96 (−18.11; +3.15)	0.40
Knee flexion (Nm)	1.84 (−1.26; +5.97)	0.20	−0.51 (−10.13; +6.43)	0.67
Hip flexion (Nm)	3.97 (−1.7; +9.84)	0.14	−0.75 (−15.1; +3.9)	0.40
Hip extension (Nm)	2 (−0.13; +5.47)	0.08	−4.15 (−16.04; +1.82)	0.40
6-MWT, m	0 (−9.5; +11)	0.96	10 (−24.7; +7.5)	0.48
Time to walk 10 m, seconds	0(−1.44; +0.4)	0.43	−0.1(−2.52; +0.32)	0.91
Time to climb 4 steps, seconds	0 (−0.42; +0.5)	0.77	−0.1 (−0.32; 0)	0.39
Time to descend 4 steps, seconds	0 (−0.35; +1.32)	0.43	−0.05 (−0.12; +0.1)	0.55
Timed up & go test, sec	0.6 (−0.4; +5.2)	0.47	−0.15 (−0.72; +0.97)	0.94
MFM-20, score	0 (−2; +2)	0.63	0 (0; +1)	0.33
**Respiratory studies**				
CVF seated (%)	−0.3 (−4; +3)	0.24	0 (−6; +5.77)	0.73
CVF lying (%)	0 (−6.2; +7.62)	0.73	9 (−2; +12)	0.13
**Muscle MRI**				
qMRI Thighs	1.79 (+0.2; +2.4)	**0.001**	−0.11 (−0.82; +0.4)	0.57
qMRI Paraspinal	−0.03 (−2.23; +0.73)	0.34	0 (−1.25; +1.89)	0.77
**Patient-reported outcomes**				
Activlim	33 (+1; +59)	0.81	67.5 (+37.5; +70.2)	0.31
SF36	1.8 (+0.67; +10.73)	0.22	3.28 (+24.84; +10.12)	0.89
INQoL	−2.9 (−5.5; +0.73)	0.17	−4.3 (−8.2; +1.1)	0.31

Median value and 25th–75^th^ percentiles are shown. Paired Wilcoxon signed rank test was used to find out whether the differences observed between baseline and year 1 evaluation were statistically significant. Adjustment for multiple comparisons using Bonferroni correction was applied; p was considered significant if lower than 0.002.

**Table 5:** Fat fraction progression in every thigh and trunk muscle after one year of follow-up in symptomatic and asymptomatic patients.

	Symptomatic treated patients (baseline vs. 12-month follow-up)	*P* value	Asymptomatic patients (baseline vs. 12-month follow-up)	*P* value
*Rectus Femoris*	0.44 (+0.02; +2.22)	0.04	−1.04 (−1.94–0.19)	0.13
*Vastus Lateralis*	1.4 (+0.01; +2.99)	0.009	−0.55 (−1.3; +0.22)	0.24
*Vastus Medialis*	0.22 (−1.04; +2.8)	0.15	0.48 (−1.34; +2.03)	0.44
*Vastus Intermedius*	1.66 (+0.24; +3.21)	**0.001**	−0.54 (−1; +0.05)	0.13
*Biceps Femoral short head*	1.76 (−0.3; +2.78)	0.007	0.01 (−1.23; +2.64)	0.64
*Biceps femoral long head*	1.2 (−0.2; +3.3)	0.006	0.36 (−0.79; +0.64)	0.64
*Adductor Longus*	0.04 (−1.6; +1.24)	0.93	−0.2 (−1.62; +1.35)	0.50
*Adductor Magnus*	0.51 (−0.06; +1.66)	0.053	0.3 (−1.79; +1.03)	0.57
*Sartorius*	1.03 (−0.85; +2.34)	0.08	−0.97 (−1.38; +1.13)	0.38
*Gracillis*	−0.11 (−2.44; +1.79)	0.73	−0.71 (−1.79; +0.77)	0.44
*Semitendinosus*	1.17 (0; +3.22)	0.02	−0.45 (−1.9; +0.77)	0.59
*Semimembranous*	0.6 (−0.61; +3.15)	0.10	1.82 (−0.16; +3.21)	0.02
Total thigh	1.79 (+0.2; +2.4)	**0.001**	−0.11 (−0.82; +0.4)	0.57
Paraspinal	−0.03 (−2.23; +0.73)	0.34	0 (−1.25; +1.89)	0.77
Psoas	0.97 (+0.06; +0.95)	0.02	−0.91 (−3.08; +2.71)	0.67

Median value is shown with 25th–75^th^ percentiles in brackets. Paired Wilcoxon signed rank test was used to find out whether the differences observed between baseline and year 1 evaluation were statistically significant. Adjustment for multiple comparisons using Bonferroni correction was applied: p was considered significant if lower than 0.004.

**Figure 3:**
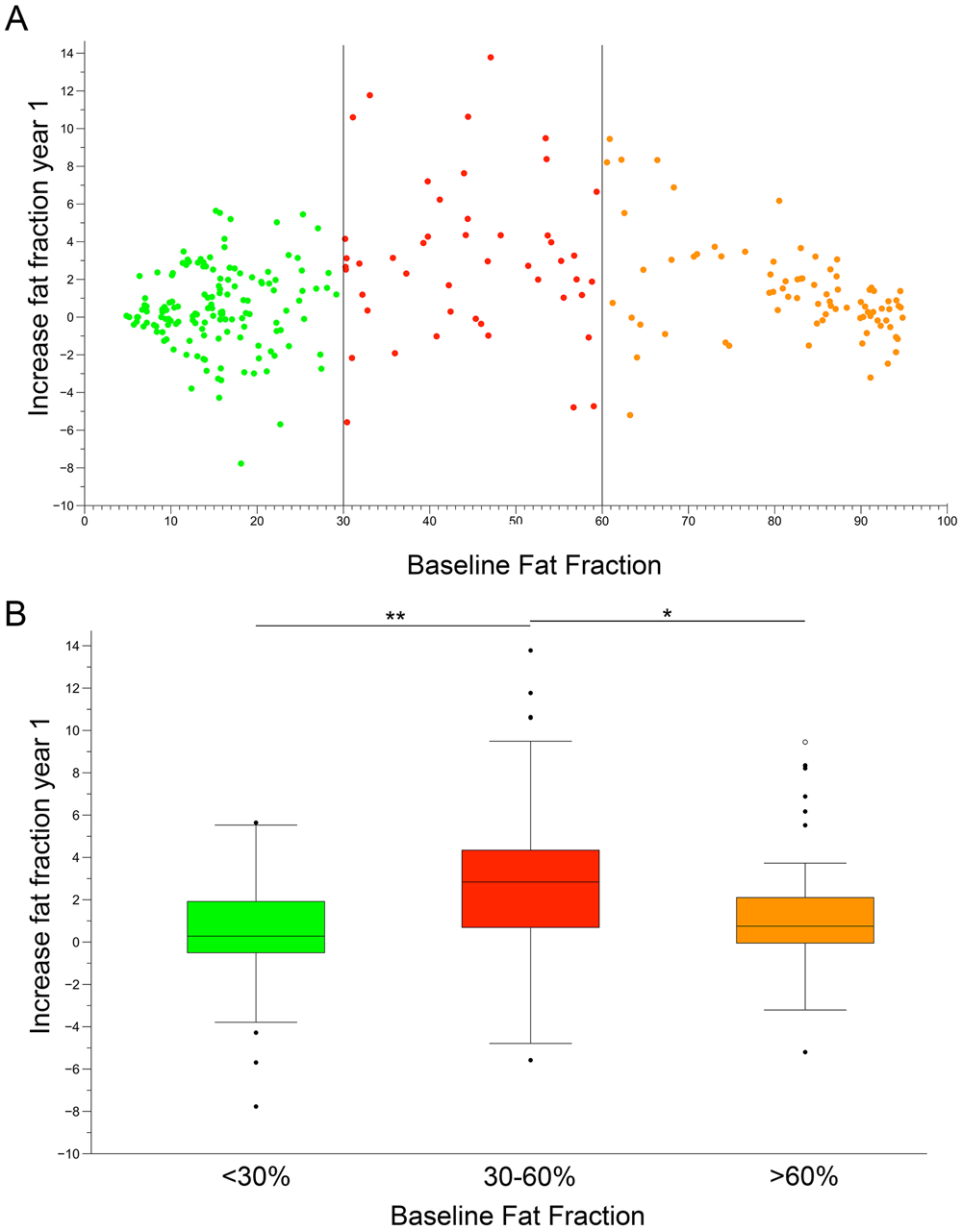
Yearly progression of fat replacement in individual muscles of symptomatic patients. (**A**) Each dot represents fat fraction calculated in a single muscle. Vertical lines divide muscles based on baseline fat fractions: low (green dots, 0–30%), intermediate (red dots, 30–60%) and severe (orange dots, 60–90%). (**B**) Increase in muscle fat replacement related to baseline fat fraction. The box plot includes the 25^th^–75^th^ percentile, the mid lines indicate the median, bars correspond to the 5^th^–95^th^ percentiles. Black dots are outliers. Kruskal-Wallis test, *P < 0.05, **P < 0.01.

We studied whether demographic or clinical factors such as sex or age at the start of ERT were associated with the increase of thigh FF at year 1. We only found a significant correlation between baseline thigh FF and increase of thigh FF at year 1 (Spearman test p = 0.024; ρ = 0.48). The thigh FF value in a single patient is an average value of fat present in muscles at different stages of involvement, from mild involvement to complete fat replacement. It has recently been shown that the basal status of muscles could influence response to treatment and consequently their increase in FF^21^. For this reason, in an exploratory post-hoc analysis, we decided to study all muscles as separate variables. We classified muscles as having mild fatty infiltration when FF at baseline was lower than 30%, moderate infiltration when FF at baseline was between 30 and 60%, and severe infiltration when FF at baseline was higher than 60%. There were significant differences in the increase in FF between the three groups after one year (Kruskal-Wallis, p < 0.0001, Fig. 3). We observed that FF in muscles with less than 30% FF at baseline increased less than muscles with FF between 30 and 60% after one year (median +0.28% vs. +2.84%, p < 0.0001). In a similar way, FF in muscles with more than 60% FF at baseline increased less than muscles with FF between 30 and 60% during the same period (median +0.75% vs. +2.84%, p = 0.004).

Muscle FF did not change significantly in asymptomatic LOPD patients. The median change in thigh FF in this group was −0.11% (Paired Wilcoxon signed rank test, P = 0.57). We did not find significant changes in muscle function tests in this group of patients after the one year follow-up (Table 4).

## Discussion

In the present study we show that quantitative muscle MRI using 3-point Dixon is a useful tool for monitoring disease progression in patients with late onset Pompe disease. qMRI was more sensitive to the changes over a period of one year than other more conventional muscle tests. We analyzed a large cohort of LOPD patients including symptomatic treated patients and asymptomatic patients with hyperCKemia. Our results support the addition of qMRI to the protocol currently used to follow up LOPD patients. In the case of treated patients, qMRI could be useful for analyzing treatment response. For asymptomatic patients, meanwhile, an increase in fat infiltration detected by qMRI, before clinical symptoms are evident, should be taken into account when considering ERT or a closer follow-up of progression.

Hereditary myopathies are characterized by slow progression over the years. Skeletal muscle is replaced by fat and fibrotic tissue leading to muscle weakness^22^. However, annual changes in muscle strength and function can be subtle and difficult to quantify, and it is not uncommon to find a heterogeneous rate of progression among patients. For these reasons, establishing the progression rate of hereditary myopathies is a difficult task. This difficulty needs to be addressed, especially at present, when several drugs designed to slow down the progression of these diseases are being tested in clinical trials^23^. To keep up with these therapeutic advances, the neuromuscular field needs reliable biomarkers to detect subtle changes in muscle structure and function^24–26^.

qMRI has recently been suggested as a useful tool for following up patients with various neuromuscular diseases^13,15,18,27,28^. We analyzed the progression of muscle fat infiltration in patients with LOPD over a period of one year using a qMRI sequence, the 3-point Dixon, which has been reported to be a valuable outcome measure, reproducible and more sensitive to change after a short period of time than muscle function tests^17^. Our results agree with this previous observation. However, in our opinion, 3-point-Dixon has some limitations. Analysis of the images is still performed by hand as ROIs are manually drawn on muscle sections, making the evaluation of images a time-consuming task that requires a high degree of expertise and patience. Software that can automatically calculate fat fraction on an entire slice stack has been developed, but has not yet been applied to neuromuscular disorders^29^. As fat infiltration is not homogeneous through all the muscle, several slices should be analyzed to calculate median fat infiltration. Moreover, the analysis of a new set of images obtained from the same patients after a period of time requires internal anatomic landmarks to facilitate the recognition of slices that were previously quantified.

This is the largest prospective study using qMRI in LOPD patients reported so far. We analyzed 22 LOPD patients treated with ERT in a wide range of clinical situations, from wheelchair-bound patients requiring ventilation, to patients with slight weakness and no major problems in daily activities^30,31^. Two reports including longitudinal data of qMRI imaging from LOPD patients have already been published. Dr. Carlier and collaborators performed a retrospective analysis of 14 LOPD patients treated with ERT and found a significant increase of 0.9% in FF of all lower limb muscles during a one year period^32^. The EMBASSY study provided follow-up data for 16 LOPD patients using muscle function tests or repeated muscle biopsies^33^. Five of the 16 patients were also followed up using qMRI which included 3-point Dixon. After six months, a mean increase of 0.6% in FF of all leg muscles was observed. However, both studies analyzed lower leg muscles, such as *tibialis anterior* or *soleus*, that are commonly less involved in LOPD, probably reducing the final average increase in FF observed^19,20^.

Our study also adds new information about the natural progression of pathology in Pompe disease. 3-point Dixon imaging quantifies fat infiltration in skeletal muscles, which is a common pathological change observed in neuromuscular disorders. In the case of LOPD, the earliest pathological change is the accumulation of glycogen inside lysosomes in the sarcoplasm^34^. The progressive addition of lysosomes has two consequences. On the one hand, there is dysfunction in the contractile properties of the myofiber. On the other, the cellular autophagy process is impaired, leading to an accumulation of debris inside the muscle fiber^35–37^. Eventually, the muscle fiber degenerates and dies, and is replaced by fat, which is detectable using muscle MRI. ERT has been shown to reduce the accumulation of glycogen in the sarcoplasm and reactivate cellular autophagy, reducing muscle fiber loss^37^. We therefore argue that 3-point Dixon could be useful in following up the progression of muscle fiber loss and fatty replacement in LOPD, which is the final consequence of glycogen accumulation in lysosomes. However, 3-point Dixon imaging does not identify glycogen, which would be very useful in following up asymptomatic patients for whom muscle fiber necrosis has probably just started or is minimal^38^. MRI sequences to detect glycogen in human tissue have been developed, including (13)C-MR spectroscopy, chemical exchange saturation transfer imaging or proton MRS. However, this technology is not routinely available on standard clinical scanners^39^. MRI sequences commonly used in clinical centers include T1, 3-point Dixon, T2 and STIR^40^. An increase in the STIR signal in muscles from LOPD patients has recently been described, with the suggestion that this is related to the presence of water molecules retained by glycogen^41^. Nevertheless, this increase was not detected in all Pompe patients. In a similar way, an increased T2 signal has been described in some muscles in Pompe patients^32^. Although the increase in STIR signal has been related to accumulation of water or edema, the increase in T2 signal is not specific and can be observed in muscle fiber necrosis, inflammation, or presence of free water^42,43^. We therefore still need MRI sequences capable of identifying glycogen that can be used in clinical settings, and future research should focus on this area.

We detected an average increase in FF of 1.7% in symptomatic ERT treated patients after one year of follow-up. In contrast, no functional changes were observed in the same period of time. The increase in FF detected in ERT-treated patients is probably not enough to produce a functional change. However, the progressive increase of FF could result in a deterioration of muscle function after several years, as has been recently described in patients treated with ERT for more than 5 years^11^. It would be very valuable to identify the ratio of FF increase in non-treated symptomatic LOPD patients. One of the limitations of this study is that we had no control group including symptomatic non-treated patients, because ERT usually starts soon after patients develop muscle symptoms.

The factors that could influence the response of LOPD to ERT are still not known. The majority of muscles had a slow, continuous progression despite ERT treatment, which is reminiscent of that observed in other slow, progressive muscular dystrophies. We observed that those muscles which were less infiltrated at the basal evaluation were the ones that progressed the least. This finding has also been reported by other groups studying Pompe disease. In our opinion, this may be due to the fact that in muscles with a certain degree of degeneration, autophagic vacuoles accumulate, impairing the transport of the enzyme from the sarcolemma to the lysosomes and probably reducing the effect of ERT^35–37^. However, this rate of progression has also been described in patients with facioscapulohumeral muscle dystrophy^28^, suggesting that a certain amount of baseline damage in skeletal muscles can be associated with a more rapid progression of the disease. If this were confirmed, early treatment of patients could result in a better and longer-lasting response to drugs, although this should be further investigated in a prospective clinical trial^21,37^. In any case, we think qMRI may play an important role in the early detection of fatty infiltration in muscle, which would suggest following up asymptomatic patients more closely.

Based on our results, we conclude that qMRI is a very efficient tool for demonstrating the muscular condition of Pompe patients from the morphological point of view, and to monitor symptomatic and asymptomatic LOPD patients treated or not with ERT in daily clinics. However, its utility in detecting muscle degeneration in short therapeutic trials may be questionable.

## Methods

### Study design and participants

We are conducting a prospective observational study following up LOPD patients at the Hospital de la Santa Creu i Sant Pau (HSCSP) in Barcelona. This comprises annual evaluations, including muscle function tests, spirometry, quality of life scales and quantitative muscle MRI. This study is registered in ClinicalTrials.gov with the identifier NCT01914536. The HSCSP ethics committee approved the study and all participants signed an informed consent form. All study procedures were performed in accordance with Spanish regulations.

Inclusion criteria for the study were: (1) Diagnosis of LOPD based on recommendations recently proposed by the European Pompe Consortium; reduced enzymatic activity in leukocytes, fibroblasts or skeletal muscle and/or the presence of two mutations in the *GAA* gene following the diagnostic^44^; (2) No contraindications to MRI; (3) Symptoms of muscle weakness starting after the age of 18; (4) Willingness to complete all muscle function tests, respiratory assessment and patient-reported outcomes measures.

All patients were studied by two physiotherapists (I.B. and I.P.) with considerable experience in neuromuscular disorders at HSCSP in Barcelona. The physiotherapists evaluated muscle function using the following tests: the 6 minute walking test, time to walk 10 meters, timed up-and-go test, time to climb up and down 4 steps, and the motor function measure-20 item scale (MFM-20)^45^. Muscle strength was studied using both the Muscle Research Council scale (MRC) and hand-held myometry. ACTIVLIM, INQoL and SF-36 were used as patient-reported outcome measures^46^. We obtained forced vital capacity, both seated and lying down, using the Carefusion Microlab ML 3500 MK8 spirometer (Carefusion, Yorba Linda, CA, USA).

Patients were classified as symptomatic or asymptomatic. We considered a patient symptomatic when muscle weakness was found in clinical examination using the MRC scale, or when Forced Vital Capacity was at less than 85% of the normal values.

### Muscle imaging

All patients were examined in a Philips Achieva XR 1.5 Tesla located at HSCSP. We used the same positioning protocol for all patients: a supine position with the legs stretched out, the patella facing upwards and the ankles in a neutral position.

Axial 3D 3-point Dixon images were acquired with the following acquisition parameters: TR/TE = 5.78/1.8, 4 ms, flip angle = 15°, FOV = 520 × 340 × 300 mm, voxel size = 1 × 1 × 3 mm for thighs and FOV = 520 × 320 × 200 mm and voxel size = 1.3 × 1.7 × 5 mm for the lower trunk. The acquisition time was 45 minutes per patient.

Two investigators (S.F-B. and J.D-M.) analyzed 3-point Dixon MR images using the PRIDE (Philips Research Image Development Environment) tool. ROIs were manually drawn on five slices of the following muscles: *rectus femoris*, *vastus intermedius*, *vastus lateralis*, *vastus medialis*, *adductor magnus*, *sartorius*, *gracilis*, *semitendinosus* and *semimembranosus*; on three slices of *biceps femoris long head*, *biceps femoris short head* and *adductor longus*; and on two slices for *psoas* and lumbar paraspinal muscles. We used anatomical landmarks to ensure consistency between baseline and year 1 analysis: we took the last slice in which the femoral head was observed as a reference, and analyzed muscles every 5 cm along the entire thigh.

For every ROI, the total area and area covered by fat were calculated automatically using the PRIDE tool (Fig. 1A,B). The fat fraction coefficient was defined as $fat/fat+water$ where *fat* and *water* were the image intensity values over the ROI for the fat and water Dixon images respectively. From those two parameters, and assuming that water content corresponds mainly to muscle, fat and muscle area were estimated. Accumulative values across all slices were also computed and, as a final index, muscle Fat Fraction (FF) was calculated as follows: FF = (muscle fat area × 100)/muscle area. Once we obtained the values from every muscle we calculated thigh fat fraction as follows: thigh FF = (Sum of fat area of all thigh muscles × 100)/sum of muscle area of all thigh muscles).

The average time to quantify fat fraction in 3-point Dixon images was 60 minutes. A high degree of reliability was found between investigators. The IIC coefficient was 0.982 with a 95% confidence interval from 0.977 to 0.987.

### Statistics

We used non-parametric tests for the statistical analysis of the variables. We used the Mann-Whitney U test to investigate whether there were significant differences between variables between groups (symptomatic vs. asymptomatic). Agreement between fat fraction measurements in quantitative muscle MRI, taken by two different investigators, was measured using the Intraclass Correlation Coefficient (IIC). We used the paired Wilcoxon signed rank test to investigate whether there were significant changes in motor function tests, spirometry, quality of life scales and the fat fraction measurements obtained with qMRI between the baseline and year 1 visit. We used Spearman’s rank correlation (coefficient reported as ρ) to investigate whether there was a correlation between the results of the muscle function tests, spirometry, quality of life scales and the thigh fat fraction obtained using qMRI. As we ran multiple correlations, a Bonferroni test was performed to avoid type 1 errors. An ROC curve was performed to study whether thigh fat fraction was able to differentiate between symptomatic and asymptomatic Pompe patients with high sensitivity and specificity. The results of all statistical studies were considered significant if P was lower than 0.05. Statistical studies were performed using IBM SPSS® Statistics software version 21.

The datasets generated during the current study are available from the corresponding author on reasonable request.
